# Practical Illustrations of Typhus, &c.

**Published:** 1817-02

**Authors:** 


### PART II.

### COMPREHENSIVE ANALYTICAL REVIEW OF MEDICAL LITERATURE.

"Tros, tyriusve, nobis nullo discrimine agetur."

### *Practical Illustrations of Typhus, &c.*
### By JOHN ARMSTRONG, M. D.

( Concluded from page 45. )

WE know not which to admire most—Dr. Armstrong's inimitable delineations, or his judicious modes of treatment, in the Proteian forms of typhoid fevers. Of the *former* we have given a sufficient specimen in our last Number; we now proceed to the *latter*, with not less confidence that our readers will enjoy, in an equal degree with ourselves, the intellectual banquet. Would to Heaven, that, as public caterers, we could always set such delicious fare before our guests!

*Treatment of the simple typhus.* Unhappily, in fevers of this description, the physician is seldom summoned till "a dangerous combination of symptoms renders the success of the best measures uncertain." As the symptoms vary according to the stage, and to the duration of that stage, it shews the necessity of ascertaining with accuracy the progress of the disorder, since the remedies proper at one period will be improper at another. Were this attended to, what controversies would be avoided—what jarring contrarieties might probably be reconciled!

*Ab initio febris,* rest should be enjoined. It is in the first stage that, by "*retrocession of the blood from the surface, and certain internal accumulations, the general balance of the circulation is disturbed.*"

"The practitioner must not here be deterred by the appearances of *debility* from the use of evacuants, for they are not only safe but highly salutary at this time, when the system is merely *oppressed by a kind of preternatural burden*, and not really in a state of exhaustion." 101.

Antimonial emetics are serviceable when the fever is of the least complicated form, as they improve the condition of the skin, respiration, and pulse. The bowels are next to be moved both by purgatives and glysters. As torpor very generally pervades the intestinal canal, brisk cathartics are necessary;

"Nor need any risk be apprehended from three, four, or even more, copious motions during the day; for instead of weakening the patient, they will renovate his powers, lighten the system of the load which weighs it down, and contribute to restore the circulation to its healthy equilibrium." 102.

These means alone will sometimes break the febrile catenation *in embryo*, and always shorten its duration and lessen its danger. The want of preternatural heat of the surface now forbids the cold affusion; "but the warm bath is a safe and efficacious remedy, and, with the means before mentioned, has considerable effect in equalizing the circulation." Tepid barley water or thin gruel should now form both the beverage and the diet. The diffusible stimuli are in general inadmissible at this period, as they render the subsequent excitement more violent.

The temperature of the apartment is recommended by our author to be kept in this stage at 56 or 60° of Fahrenheit. A lower temperature may induce local determinations, particularly to the chest.

*Second stage of simple typhus.* As dry burning heat of the skin is amongst the most conspicuous symptoms, Dr. Armstrong makes some very judicious strictures on the principles and practice of the illustrious Currie. They are applicable to the stage of excitement, but not that of collapse, though in the *latter*, the conditions are occasionally present, which, according to his ideas, require the cold affusion. During the 1st, 2d, and 3d day of the stage of excitement, Dr. A. has seen simple typhus entirely extinguished by the affusions of cold water; and failing to effect this, they have refreshed the patient, lessened the fever, and insured a favourable issue. From the 4th day of this stage till the period of collapse, tepid affusions or bath of 94 or 96.

"It is in this stage that purgative medicines are so exceedingly useful; and they ought to be exhibited every day, either a little before or after the application of the warm or cold affusions," 110.

Dr. A. thinks, and we fully agree with him, that the beneficial influence of purgative medicines extends much farther than the mere removal of fæcal matter from the intestinal canal, as was the opinion of Dr. Hamilton.

"Is it not exceedingly probable (says Dr. A.) that they have another and even more salutary effect, in restoring healthy secretion, and removing irregular distributions of blood from the head, liver, and other vital parts?" 112.

That the doctrines of debility and putrescency have yet a considerable hold on a certain portion of medical society, we have often stated in this Journal. Dr. A. confirms this statement.

"Strange as it may appear (says he) it is still the custom of *many practitioners* to pour in large quantities of wine indiscriminately throughout all the stages of the genuine typhus. If, by any chance, the energies of the constitution should finally prevail against both the disease and this injudicious treatment, the recovery is falsely attributed to the wine, and thus a most dangerous error is, at once, both propagated and respected: — an error by which an immense number of febrile patients has been destroyed." 114.

It is needless to say, that food is out of the question during the stage of excitement in this and all other fevers.

"Milk mixed with two parts of water and a little arrow-root, milk whey, barley water, thin gruel, and the like, will answer every purpose of sustaining the powers of the system without exciting the circulation." ib.

In the third, or stage of collapse, we must be more cautious in the administration of purgatives. Two moderate dejections daily will be sufficient, unless evacuations have been neglected in the beginning, when an extraordinary accumulation of fæces is often found to exist in the ulterior periods, occasioning great determination to the head; oppression of the sensorial powers; prostration of strength; flushed face; suffused eye; delirium, &c. In these cases, abstraction of even a small quantity of blood would be very hazardous; whereas full purging with calomel and jalap, supporting the strength at the same time with moderate doses of wine, will often produce the most agreeable change. In the last stages of typhus, frequent, small, loose, fœtid stools, occasionally mixed with slime and blood, are not seldom indicative of loaded bowels, the consequence of inattention in the earlier stages. On the contrary, if patients have been kept in very close apartments, even with attention to intestinal evacuations, it will be occasionally observed, that, on the approach of the last stage, frequent, copious, bloody stools are passed without any offensive odour, at the same time that dark petechiæ begin to shew themselves upon the extremities, becoming more numerous, and spreading over other parts of the body. Here evacuations increase the effusions of blood, and suddenly depress the vital powers. Danger is great here, whatever plan we pursue—

"—but the free admission of fresh air, the liberal allowance of lemon-juice mixed in a little Madeira wine and water, with small repeated doses of opium and aromatics, are the means on which most reliance may be placed." 120.

"It is in the stage of collapse, that the principles laid down by the philosophic Dr. Currie may be deceptive, with regard to the application of cold water. For it not unfrequently happens, that a glow of heat is diffused over the surface, and if the thermometer be applied, a temperature somewhat above natural will even be manifested, which may continue for one or two hours together, though it is seldom longer stationary at one time. Yet if the patient be narrowly examined, some parts of the body will be found rather cooler than others, and symptoms of general relaxation will be pretty evident. At this period, neither the cold nor the tepid affusions can be applied to the whole surface without great risk, though partial ablutions of the hands, face, breast, and feet, with cool or warm water, are often highly refreshing, as are likewise the temporary admission of cool air, and very small draughts of any cool liquid; such being the difference between the partial and general application of the same or similar means. If a man were excessively fatigued by a long journey on a summer's day, the dashing of three or four gallons of cold water over his naked body would almost chill him to death. But let his hands, face, and feet, be quickly washed in cool water, give him a little cool wine and water to drink, and you diffuse new life throughout his languid frame: something similar may often be remarked, where partial ablutions, and small portions of cool drinks, have been employed, in the last stage of the simple typhus." 124.

In the last stage, wine cautiously administered, at first in small quantities, watching its effects, will be often necessary. Madeira is perhaps preferable to every other, being grateful, and at the same time remaining lightly on the stomach. It should not be given undiluted. Dr. A. recommends a mixture of one part of Madeira to four or five of milk, as an excellent drink and diet combined. He thinks about two-thirds of a pint may be considered sufficient of wine in the twenty-four hours, for an adult who has lived temperately. Strong stimulants at this time—

"—may be compared to the violent lashing and spurring of an exhausted animal, in order to keep it in motion; for in both cases the vital principle would immediately be raised by the applied powers, but falling in proportion to each preceding excitement, it would be rapidly and irretrievably sunk by the frequent repetition of such treatment." 126.

When wines disagree, malt liquor will sometimes sit easy on the stomach.

Dr. A. notices a peculiar modification of the simple typhus, which occurs in feeble hysterical women and constitutions broken down by intemperance. It is now and then attended with a wild, almost maniacal delirium.

"The chief remedies for it are mild purgatives, the warm affusions, and small frequent doses of opium, combined with calomel, as an alterative."

Dr. A. justly observes, that the stage of collapse sometimes rapidly supervenes in drunkards, who, on that account, should have an earlier and freer allowance of stimulants.

Dr. A. thinks, that in this species of typhus venæsection will seldom be necessary where the foregoing rules are observed. But whenever topical determinations appear, then local or general blood-letting, of course, will be proper, together with blisters, &c.

*Treatment of the inflammatory typhus.* There is no doubt but that indiscriminate blood-letting, without regard to the period of the disease, the age, the habit, or the constitution of the patient, may, and often does much mischief:

"But, on the contrary, if it be employed with discretion at an early period of the inflammatory or congestive varieties of the complaint, it may be proved to be an instrument of the greatest utility, effecting what no other can so well effect—the preservation of the structure of the main parts in the living machine, when endangered by preternatural determinations or accumulations of blood. Forty years ago, purgative medicines were prohibited, through the prevalence of false hypotheses; but happily their efficacy is now universally acknowledged: and before forty years more shall have elapsed, the efficacy of blood-letting, I doubt not, will be as firmly established in certain varieties and stages of most contagious fevers." 132.

"During the rise and progress of the fatal doctrines of debility and putridity, the lancet was condemned in many fevers, and by authors who, with a singular inconsistency, continued to commend the sagacity of Sydenham. But the speculations of Cullen, and other men of genius, which have so long obscured our pathological views are at length passing away, like clouds, before the spreading light of more favoured times, and we may reasonably hope will soon entirely disappear from the horizon of the medical world.' 133.

" Probably the numerous and very excellent treatises, published by the practitioners in warmer climates, where fever presents a more ardent aspect and is attended with higher excitements, and where the efficacy of blood-letting has staggered even the faith of some staunch Brunonians, have powerfully contributed to produce the recent changes in many important points of opinion and practice. Yet, with whatever present disadvantages it may be attended, the lapse of a few years will enable the physicians of Great Britain to appreciate and apply venæsection as correctly as other evacuants. The grand principle of early depletion may be properly extended to almost all acute fevers, idiopathic and symptomatic; although it cannot always be carried so far in the former as the latter, nor perhaps in temperate as in tropical regions." 134.

" It is in the acute species of inflammation, sometimes commencing on the first, second, or third day of the second stage of typhus, for which, provided it be seated in a part of vital importance, copious venæsection is indispensable. At an early period of such cases, the strength is depressed, but not subdued; and, as the depression is then principally the effect of the topical disorder, venæsection, by diminishing or removing that disorder, diminishes or removes the load which impeded the vital functions; and the strength, compared before and after the operation, is therefore increased, instead of being lessened; a fact which I have frequently noticed. But it must never be forgotten, that general blood-letting is only advantageous, or even admissible, in the beginning or acme of the acute inflammation, because when it has existed for a few days, it is almost invariably combined with universal exhaustion; and venæsection will then hardly ever remove it, but contribute to precipitate the patient to the grave, by its powerful impression upon the whole system. When called, therefore, to any case of typhus, complicated with an acute inflammation, the practitioner should ascertain, as precisely as possible, the duration of the latter, and the state of the general system. If the topical affection has been but of short continuance, and the vigour of the constitution be merely weighed down, and not really exhausted, let him discard the fears associated with false doctrines, and promptly abstract blood, according to the seat and extent of the inflammation, and till the local pain and general oppression be relieved. But if, on the contrary, the topical affection has continued for some days, and there are symptoms of a present or an approaching collapse, let not the evidences of any local derangement, induce him to hazard general venæsection, as he values the life of the patient and his own reputation. I have been often consulted in typhus, at the critical moment when the inflammation had so far advanced, as to render the propriety of decided practice very questionable, and yet not entirely to preclude the employment of depletion. In such instances of uncertainty, it may be assumed as a principle, that local is preferable to general blood-letting; and, in conjunction with blisters and purgatives, it will sometimes surpass the expectations of the practitioner. There are circumstances which will even justify the simultaneous employment of local blood-letting and diffusible stimulants in typhus; as, for example, when the stage of collapse approaches, and the head or chest, from the previous one of excitement, has become oppressed with an engorgement of blood, which is rapidly overpowering the vital energy. In such lamentable instances, although blisters, laxatives, and mercurials, may be conjointly serviceable, the immediate chance of relief is from the local abstraction of blood, by leeching or cupping, and the exhibition of wine; the first with a view to relieve the topical accumulation, and the last to support the system under the evacuation. What was formerly said about the combination of purgatives with cordials, in the last stage of the simple typhus, may tend to illustrate the union of these seemingly inconsistent, but sometimes efficacious means, against the conjoint use of which the ingenious Dr. John Brown has so indiscriminately protested." 137.

Dr. A. justly remarks, that although both general and local bleeding is necessary in typhus, yet these evacuations cannot be borne to the same extent as in pure inflammations. Nothing can be of greater consequence than to note accurately the stadia of acute diseases, otherwise the disputes may be endless about their modes of treatment, which must correspond to the leading phænomena of each stage. In all fevers of an open character, there are two grand stages; one of excitement, another of collapse:

" It is in the former that depletion is so excellent, while it is always dubious, and often extremely dangerous, in the latter. In violent cases, the stage of excitement soon passes away, and then come those malignant symptoms, *as its effects*, about which so much has been written, and which, viewed independently of the preceding one, have contributed to mislead so many pathologists and practitioners."

Convinced of the great importance of making a strong and early impression on inflammatory typhus, Dr. A. directs, where there is suspicion of acute inflammation of any viscus, that blood be drawn till faintness insure subsequent syncope. The latter should be induced with as little loss of blood as possible; hence a large orifice and perpendicular position are best adapted to effect the desired purpose. Dr. Armstrong has in general found one or two good bleedings sufficient at the beginning, when properly assisted by other means.

"In fact (says our author) I am no advocate for large repeated abstractions of blood in the inflammatory typhus, but rather trust to one or two well-timed and moderate attempts by the lancet, and then place my chief reliance upon saturating the system with mercury.

"Perhaps few physicians in this country have given calomel with more freedom than myself in febrile disorders. Having imbibed an early and strong bias, that its whole efficacy depended upon its purgative operation, I remained sceptical for a long time as to its possessing any other power in fevers. But cautious and reiterated observations have at last convinced me, that, next to venæsection, it is one of the most powerful anti-inflammatory agents with which we are acquainted. It will be found, that few patients perish in inflammatory diseases, where ptyalism is clearly established. In general terms, inflammation may be denominated a loss of balance in the circulating system, and *calomel, having a direct power in equalizing the circulation*, is a most suitable remedy for that affection. But the ordinary mode of administering it will do little good, nay will often tend to bring it into discredit. Before its exhibition in the inflammatory typhus, blood should always be drawn, and the bowels freely opened. Then, without any loss of time, it should be administered in at least eight grain doses, combined with about a grain of opium, and repeated three, four, or five times in twenty-four hours, until the mouth be obviously affected. In cases of more than ordinary violence, I have often given it in scruple doses; and so far from having had reason to regret this practice, it has generally proved most beneficial, by rapidly saturating the system, and thus striking at the very seat of the inflammation. The combining of moderate doses of opium with large ones of calomel, has most frequently an admirable influence, not only in causing a complete change of action in the system, but in promoting an universal warm perspiration, which tends speedily to remove every trace of the internal disorder." 142.

We hope that such a declaration, from such a man as Dr. Armstrong, will have some effect in checking those mawkish complaints against mercury in febrile states of the system, which we every day hear from people whose pusillanimity never permitted them to give it a fair trial; but who, from the long habit of talking nonsense, absolutely at last believe that they are speaking truth!

Where evident determinations of blood take place to particular organs, general blood-letting should always precede local; because the *former* will arrest that violent action of the heart and arteries, by which the *latter* is partly maintained. Dr. A. confirms a remark of Dr.

Stoker, that, in almost every instance of fever, the temporal arteries will be easily made to bleed freely, since the arteries leading to the head, in general, are found more distended and pulsating than natural. Dr. A. truly observes, that we should neither confide entirely in one powerful measure, nor in a combination of secondary means:—" but rather to employ from the first, the most powerful antiphlogistic agents, successively or together, that their influence may be so exerted as to produce a complete change of action in the circulation with the least possible loss of time." In this respect, Sydenham himself was deficient, trusting to venæsection in the first instance, and inert means afterwards.

" The free exhibition of purgative medicines, immediately after venæsection, is one of the greatest improvements in modern medicine, as it respects the cure of acute fevers; and if, to the agency of those two means, that of calomel, as alterative, be added, we give a summary of what might *always* be attempted, not only in the inflammatory typhus, but in all the varieties of acute visceral inflammations. P. 146.

Blisters *after* evacuations are also serviceable. Dr. A's experience in the cold affusions does not enable him to speak decidedly on the subject. Where there are unequal distributions of blood, with unequal degrees of heat on the surface, the cold affusions cannot safely be ventured on; but the warm bath, after bleeding and purging, has the power of equalizing the circulation, by inducing a flow of blood towards the surface.

In hepatic, gastric, and other abdominal inflammations conjoined with typhus, Dr. A. properly depends much on purgatives, the large intestines being first evacuated by glysters, containing a large quantity of fluid; and where a state of irritability remains, opium cautiously administered.

Dr. Armstrong enters into an enumeration of the means necessary to be employed in certain diseases accompanying typhus, such as dysentery, erysipelas, &c.

" There can be no doubt that dysentery may be cured by very different methods: by bleeding and purging, by mercurials and opium, or simply by a conjunction of cathartics with sudorifics. But amidst the numerous plans that have been recommended, it is certainly most desirable to know those upon which most confidence may be placed. Dysentery is almost always attended with an acute or sub-acute inflammation of the villous coat of the intestines, and generally with a congestive or inflammatory state of the liver. If the inflammation of the villous coat of the intestines be of the sub-acute kind, and the affection of the liver of a similar nature, daily purgation, by large doses of calomel, and moderate ones of castor oil, with occasional anodynes and the tepid bath, may certainly often answer every purpose. Yet it is invariably much better, even in such instances, to bleed moderately at the very beginning, and, the bowels having been freely evacuated, to produce ptyalism as rapidly as possible, by the administration of calomel and opium. It may be said, that many cases of dysentery have been cured without venæsection, and there is no denying the assertion. But if my observation be correct, early venæsection at once diminishes the force of the abdominal affections; renders the system much more susceptible of the action of purgatives and mercury; and, not only shortens the duration of the disease, but lessens the chances of chronic affections supervening afterwards." P. 167.

He considers, that when dysentery is accompanied by villous inflammation and hepatic congestion, early and decided blood-letting is necessary. These usually commence with oppression of the whole system; pain in the abdomen, sometimes permanent, sometimes periodical; short, hurried breathing; quick pulse; hot skin; sense of internal heat; tenesmus; small, slimy discharges mixed with blood. If, on the second or third day, the fever ceases or signally remits, the pulse becomes quicker and smaller, the breathing more frequent, the temperature more variable, the debility greater, then the stomach will become extremely irritable, the pulse sunk, the extremities cold, and death will close the scene about the seventh or eighth day.

"On examining the body in such instances, the intestines will commonly be found distended with fœtid air—their lining in a gangrenous state, *and the liver engorged with blood.*"

In these rapid and dangerous forms of dysentery, common modes of treatment must not be trusted to. Venæsection, purgatives, calomel, opium, and blisters must be boldly employed.

"As soon, therefore, as a sufficient quantity of blood has been drawn, *a scruple of calomel* ought to be given, with one or two grains of opium. This combination not only tends to take off general and local irritation, but also to facilitate the operation of aperients, as has been already explained, in speaking of certain modifications of the inflammatory typhus." P. 169.

It will thus be seen, that the utility of scruple doses of calomel in dysentery, has been appreciated in the North as well as in the South; and we hope to hear no more hypothetical objections made by timidity and prejudice against this valuable medicine.

"But to return to the treatment of dysentery: at the same time that the blister is applied, *calomel should be boldly exhibited*, and continued afterwards, with small doses of opium, that its specific effect may be established as speedily as possible." P. 170.

Again,

"Though I have considered this disease as closely connected with an acute or sub-acute inflammation of the villous coat of the intestines, and a *congestive or inflammatory state of the liver;* yet, in every instance, I believe, the functions of the skin are primarily affected, and continue disordered during the whole period of the disease; indeed, it will be found no unimportant part of the cure, to restore them to a natural condition. All the cases of dysentery which I have attended, originated from the influence of cold, under whatever form, applied to the surface. The first effect of this influence was, to produce a recoil of blood from the superficial vessels—and an internal accumulation succeeded, which apparently produced the affection of the liver and villous coat of the bowels." P. 171.

Long familiarity with the disease, personal experience of its effects, and careful observation of its phænomena, have convinced us that the views of Dr. Armstrong are founded on the immutable basis of nature and truth. Speaking of calomel and opium, Dr. A. observes, that

"The proper combination of these two medicines has a surprising effect in restoring the natural balance of the vascular system, and in promoting *a free secretion of bile, and of perspiration.* When, however, it fails in acting forcibly on the skin in dysentery, which will rarely be the case, small doses of pulv. antimonialis and camphor may be added to it with advantage; the warm bath being occasionally used at the same time, more effectually to equallize the circulation." P. 172.

In erysipelas, Dr. Armstrong recommends the plan of incisions as recommended by Dr. Hutchinson and Dr. Johnson, in the Med. Chir. Transactions and Med. Chir. Journal, and the antiphlogistic regimen in its fullest extent.

"For some years past, I have closely attended to the nature of erysipelas, and I can conscientiously declare, that I have always found it to be a sthenic disease in the commencement. Like every other acute fever, it is only asthenic in the last stage, in which a universal collapse occurs, as the mere product of preceding excitement or congestion." P. 177.

We think the following passage, though rather out of place, will be acceptable to our readers.

"There is one variety of erysipelas, which has not yet received that deep consideration from the faculty which its importance and danger deserves. It attacks infants, generally under a year old, and first appears on some part of the upper or lower extremities, leaving one place and then affecting another, till, at last, it successively travels over almost all the surface of the body. Nay, I have known two cases, where an erysipelas of this nature went twice over the whole skin, in the manner just described. This disease is often produced in infants by cold; sometimes it arises from the irritation of teething, and at other times from an improper diet and disordered bowels. It is usually attended with a considerable fever, and the secretions of the liver and intestines are very morbid, if not at its commencement, at least during its progress. The little sufferer is liable to become delirious; and, if the disease should not be early arrested, generally expires in coma or convulsions within the first three weeks. Though this is a strictly erysipelas phlegmonoides, I have not myself seen it produce those large effusions and suppurations under the integuments, which are not uncommon in the extremities of adults attacked by the disease. Whatever plan of treatment be adopted in the infantile erysipelas, success will be uncertain; yet the following has appeared to me more efficacious than any other. The primæ viæ should be freely evacuated without loss of time, first by an antimonial emetic, and then by repeated doses of calomel and castor oil; indeed, during the continuance of the complaint, copious motions should be daily procured. If the stools should have a sour smell, or become greenish soon after they have been evacuated, a little magnesia and rhubarb ought to be administered along with the calomel. Soon after the first attack, also, as many leeches should be applied as will abstract sufficient blood to induce faintness, which will sometimes prove decidedly beneficial in arresting the disorder. Although an expert surgeon may generally succeed in drawing blood from the external jugular vein, or the anterior branch of the temporal artery, in infants under a year old, yet, all the benefit which can result from bleeding, may be obtained by the proper management of leeches; which, on account of the highly vascular state of the skin, draw more blood from children than adults. Nay, in this particular instance, local is superior to general blood-letting; because by it you can make a direct and powerful impression on the erysipelas itself, and likewise induce a general change of action in the circulation, by persevering in it till faintness supervene; or, at least, till the child begin to heave at the chest and turn pale in the face. The leeches may be twice or thrice re-applied within the first four or five days, if the disorder be unusually severe. Sydenham was a great advocate for bleeding in the febrile complaints of children, and it is certainly too much neglected in our time; in fact, it is a remedy of great utility, and they can bear it well in the commencement of acute diseases, though it should never be recommended in the advanced stages, for their strength then falls under it with rapidity. When enough blood has been drawn in this infantine erysipelas, a small blister may be often advantageously applied over the stomach, or between the shoulders. If there should be any tension upon the gums, they ought to be freely lanced. The general irritation of the system must be allayed by an occasional tepid bath, or by a few drops of laudanum, under the form of an emetic." P. 180.

We must pass over a great variety of most interesting matter, in order to give a sketch of the treatment in the last variety of typhus, termed by our author the congestive. Here there is little or no intermediate stage of reaction. He considers this variety of typhus as comprehending the different characters of what some authors have been pleased to call the low, malignant, contagious fever of this country. The first stage of oppression is exceedingly short; as it affords the only period in which medical assistance is likely to be available, it is in the last degree important to distinguish it from the stage of universal collapse. All fevers may be considered in their commencement as disordered states of the circulation, generally and locally; which, if uncorrected, induce derangement of function or structure that bids defiance to remedial means. In the variety under consideration, the stage of oppression passes directly into that of collapse—disordered action being most commonly succeeded by organic lesion.

"It is in the first stage only of the highly congestive typhus, that general blood-letting is admissible with a view of relieving the local congestions, and of restoring the natural balance of the circulation. The practitioner must not fix upon a determinate quantity of blood to be drawn, but rather be guided by the effects produced. Sometimes eight or ten ounces will be quite sufficient; and at other times, sixteen, twenty, or even more, at one time, may be requisite to relieve the topical engorgements, and put the general circulation into proper play. The action of the heart is often so much overpowered in the first instance, that the blood merely trickles, or rather oozes from the punctured vessel for a considerable time, being much darker and thicker than natural. Yet, when a few ounces have been drawn, it usually flows with freedom, and becomes finally of a brighter colour. Occasionally, I have stood over a patient nearly half an hour before a stream of blood could be obtained from a vein; but at last, it gushed out in a full current, and was not so easily restrained as in ordinary cases. Many a life might be lost by binding up the arm too hastily, and therefore the operator should persevere until he knows whether enough blood can be obtained. As the engorgements are in a great measure venous in the congestive typhus, my common practice for a long time was, to order the jugular or brachial veins to be opened, conceiving that blood abstracted from one of them would sooner relieve the system than from an artery. But finding, in some cases, that a sufficiency of blood could not be obtained from the veins, I was induced to order the opening of the temporal artery; and where the operation was skilfully done, not only enough blood was procured, but a more immediate change appeared to be effected in the whole vascular system, than by venæsection simply. The circulation of the blood, it is well known, is partly carried on by the *vis a tergo*, especially that of the venous system. Now, in the highly congestive typhus, the energy of the heart and arteries is greatly oppressed from the beginning, and the *vis a tergo* being thereby diminished, the blood must necessarily flow much more languidly in the veins, and of course be less easily abstracted from them than under ordinary circumstances; while it must be equally evident, that it may at first be more readily procured from the arteries, where a comparatively freer current is still maintained. Whenever, therefore, blood cannot be obtained from the veins with a freedom or celerity commensurate to the urgency of the symptoms, the temporal artery should be opened: and though my experience has not yet enabled me to determine whether arteriotomy be *generally* more efficacious than venæsection, in congestive cases, yet, whenever the head is greatly affected, which often happens, the vessel just mentioned should always be punctured, as by so doing, I have frequently seen patients rescued from impending death.

"In great congestions, the pulse generally rises under or immediately after blood-letting; but sometimes continues oppressed, and even weaker than before. When both it and the general circulation become manifestly freer, with a warm moist skin, tepid diluents should be the only beverage recommended; yet, when it still remains oppressed, and the tide of the circulation does not return to the surface, and more especially if blood has been freely drawn, a little wine, with warm water, should be occasionally exhibited, and the patient speedily immersed in a bath, strongly impregnated with salt, about the temperature of 100° of Fahrenheit's scale. On being removed from the bath, his skin should be well rubbed with flannels dipped in warm spirits or oil, and he ought then to be laid in an aired bed, with bottles of warm water at his feet. This plan, together with a little tepid wine and water occasionally, will often promote a flow of blood towards the skin, and considerably relieve the viscera from congestion.

"If the bath can be expeditiously prepared, it has often an excellent effect, to immerse the patient in it first, and either to bleed him while he remains in it, or immediately after he leaves it; indeed, in some severe cases, I have found it impossible to get sufficient blood until a warm bath had been premised, so oppressed was the general circulation before its employment. But where the bath cannot be obtained in time, stimulating unctions must be used instead to the surface, which are sometimes very serviceable. If the functions of the skin can be properly restored in congestive fevers, recovery will generally succeed. It has often struck me, that the vapour bath would be an excellent remedy in such cases, combined with the friction of some warm or stimulating liquid, to promote perspiration. It is much to be regretted, that in common practice, a great deal of valuable time is often lost before the common bath can be made ready, but a steam one might be soon obtained by a very simple apparatus, which ought to be kept in every house.

"The means just mentioned must be promptly applied, and followed by others with as little loss of time as possible. The bowels should first be evacuated by very large stimulating enemata, and then by full doses of calomel and jalap, whilst a large blister should be applied over the region of the stomach or liver. Well knowing that the bowels are commonly very torpid, and that every moment is inestimably valuable in such rapid cases, I have generally given a scruple of calomel at first, repeating smaller doses three or four times on the first day of the attack, with the medicines above named; and when the bowels have resisted their united influence, saline purgatives have been added, that no time might be lost. The great advantages of this vigorous practice are, firstly, that you commonly obtain free evacuations in a short time, which could hardly be obtained at all under the ordinary mode, or at least but with considerable delay; and, secondly, that you most frequently obtain the alterative operation of the calomel within the first or second day, which is a circumstance highly to be desired. For a long time I overlooked one of the principal effects of calomel in congestive fevers; till at last it was forced upon me, by patients almost invariably recovering with rapidity, when ptyalism was excited. The power which calomel has in equalizing the circulation, is no where more conspicuously displayed, than in diseases of a congestive character. Before its exhibition, the skin will be cold, wan, and shrunk; the pulse feeble or oppressed, and the whole system apparently relaxed: but, as soon as the mouth is made sore from its influence, the skin becomes warm, reddish, and distended with the vigorous circulation, while the pulse is full, soft, and strong, and the general energy in a great measure restored. Anxious to procure the purgative, as well as the specific operation of calomel on the very first attack of the congestive typhus, I have seldom combined any opium with it until an action has been established on the intestines, after which small doses of opium, antimony, and camphor, may be added with considerable benefit. The liver is intimately concerned with the pathology of congestive fevers, and for the first day or two the alvine evacuations will commonly be found either as dark as tar, or whitish and slimy, though they speedily become natural when ptyalism takes place." P. 236.

Our limits are already overstepped, and we believe that we have adduced sufficient specimens of Dr. Armstrong's work, to induce every one to place it in his library. To the author himself, we beg leave to offer our sincere congratulations on his valuable performance. Excepting in the perusal and delineation of Dr. Parry's Elements of Pathology, we have not, for many years, derived so much pleasure in our analytical labours, as in the course of the present analysis. For the talents bestowed on Dr. Armstrong by Nature—for the experience gained by time—for the facts and observations treasured up by industry, he is accountable at the tribunal of humanity, and the shrine of science! The miser, who hoards up his ill-gotten wealth, and never, during his life, performs a charitable act, expiates his sins by death, which deprives him of his mammon, and sets his treasures in circulation. But the physician who descends to the grave without transmitting his quota of knowledge to posterity, commits a crime against nature and society, for which no apology can be offered—no sacrifice atone!

---

